# Enhancing the treatment potential of IL-17 antagonism in lupus nephritis: finding the right partner

**DOI:** 10.3389/fimmu.2025.1617451

**Published:** 2025-09-05

**Authors:** Yingying Gao, Guoyuan Peng, Xin Liu, Xiaochen Ren, Xiaoxiang Chen, Yifan Zhan

**Affiliations:** ^1^ Department of Rheumatology, Nantong First People’s Hospital and Nantong Hospital of Renji Hospital Affiliated to Shanghai Jiao Tong University School of Medicine, Nantong, China; ^2^ Department of Drug Discovery, Huaota Biopharm, Shanghai, China; ^3^ Department of Pharmacology, School of Pharmacy, Fudan University, Shanghai, China; ^4^ Department of Pharmacology, PharmaLegacy Laboratories Co., Ltd, Shanghai, China; ^5^ Department of Allergy, Renji Hospital Affiliated to Shanghai Jiao Tong University School of Medicine, Shanghai, China

**Keywords:** lupus nephritis, IL-17, IL-36, biologics, SLE - systemic lupus erythematosus

## Abstract

The treatment of lupus nephritis (LN) has been benefiting from biologics targeting immune cells and cytokines. IL-17 antagonists have been investigated for their potential in LN treatment, with mixed results from case reports and randomized controlled trials. Here we provide an overview of the contributions of various immune cells and kidney resident cells to LN pathogenesis and discuss relevant biologics for LN treatment. We then explore our current understanding of IL-17 and IL-17-producing cells in LN pathogenesis and examine the status of IL-17 antagonists in LN treatment. Given the limited success in clinical studies with IL-17 antagonism alone for LN, we discuss possible rational combination biologic therapies, with a focus on the potential combination with antagonism of IL-36, a cytokine family associated with SLE disease activity. Thus, emerging evidence suggests that dual biologic therapy could enhance disease control in LN.

## Introduction

Systemic lupus erythematosus (SLE) is a complex autoimmune disease accompanied by dysregulation of autoinflammatory pathways affecting multiple organs (1). Renal involvement is common, with 40% to 70% of patients developing lupus nephritis (LN). Between 4% and 28% of LN patients progress to end-stage renal disease (ESRD) (2). Among immune cells involved in SLE pathogenesis, B cells play a central role, as the production of autoantibodies against self-antigens is a hallmark of the disease, often preceding SLE diagnosis (3). Consequently, the current biologics used for SLE and LN management mainly target B cells, such as rituximab and belimumab (2). Even interferon-targeting agents (e.g., anifrolumab) approved for SLE primarily affect B cells (4).

Unlike biologics used for rheumatoid arthritis (TNF antagonists) and psoriasis (IL-17, IL-23 antagonists), the current biologics for SLE, especially with renal involvement, have shown only modest clinical benefits. While B cell-targeting therapies are common, biologics targeting other aspects of LN pathogenesis, such as IL-17 antagonists, have been studied (5). Preclinical studies demonstrate that the absence or inhibition of IL-17 reduces proteinuria and glomerulosclerosis across multiple animal models (6–8). Based on this preclinical evidence, IL-17 antagonists (e.g., secukinumab) are being considered for use in multi-refractory cases with severe kidney diseases (9, 10). Overall, positive outcomes with IL-17 antagonists are primarily derived from case reports rather than randomized controlled trials.

It is increasingly recognized that the therapeutic ceiling may have been reached with single-agent use in many difficult-to-treat autoimmune and inflammatory diseases like LN. Rational combination biologic therapy may be crucial to enhance disease control. There are ample examples of dual-targeting biologics being developed for non-oncological diseases (11–14). Here we aim to explore ways to enhance IL-17 antagonism in LN by identifying suitable partners for dual targeting. We focus on the IL-36 cytokine family, which, although less well known, plays an emerging role in SLE pathogenesis. Unlike many approved biologic targets in SLE treatment, which belong to upstream components of the cellular cascade (e.g., pDCs and B cells), both IL-17 and IL-36 can act locally and synergistically or complementarily to promote inflammation and fibrosis. It is anticipated that co-targeting IL-17 and IL-36 can directly impact renal pathology, offering benefits to patients with advanced disease.

## SLE and lupus nephritis

SLE has dysregulated innate and adaptive immunity. This aberrant immune activation drives a pathogenic cascade involving (1) sustained hyperactivation of T and B lymphocytes, (2) production of autoantibodies targeting nuclear and cytoplasmic antigens (e.g., dsDNA, Smith antigen, RNP), (3) formation of circulating immune complexes (ICs), and (4) IC deposition in target tissues—notably the glomerular basement membrane and dermoepidermal junction—which activates complement-mediated inflammation and ultimately induces end-organ damage. The outcomes of this self-reactive response manifest as systemic and localized inflammation, leading to tissue damage in numerous organs (2, 15, 16).

SLE is currently predominantly managed with non-specific immunosuppressants like glucocorticoids, cyclophosphamide, or mycophenolate and antimalarial drugs. While these drugs have managed to control the disease and reduced the 5-year mortality rates (17), they are not always effective and can have toxic side effects. The prolonged use of glucocorticoids can lead to serious side effects (18–20). Cyclophosphamide can harm gonads, leading to reduced sperm count in men and premature ovarian failure in women. Furthermore, the prolonged use of immunosuppressants could significantly increase infections that are also associated with mortality. Overall, there is great need for safer and more effective treatments for systemic lupus erythematosus.

Recent progress in biologics and small molecule development (particularly JAK inhibitor) has resulted in the creation of innovative agents that target specific pathways implicated in SLE/LN pathogenesis. Three of these agents, belimumab, anifrolumab, and guselkumab, have received FDA approval, with several additional therapeutic options currently undergoing evaluation in clinical trials. These advancements have the potential to transform the treatment of SLE. Despite recent advancements, SLE, particularly LN, continues to pose a clinical challenge for both patients and healthcare providers. As such, effective and safe biologics and small molecule drugs are still required.

## Immune abnormalities in LN and targeted biologics

**Figure 1:**
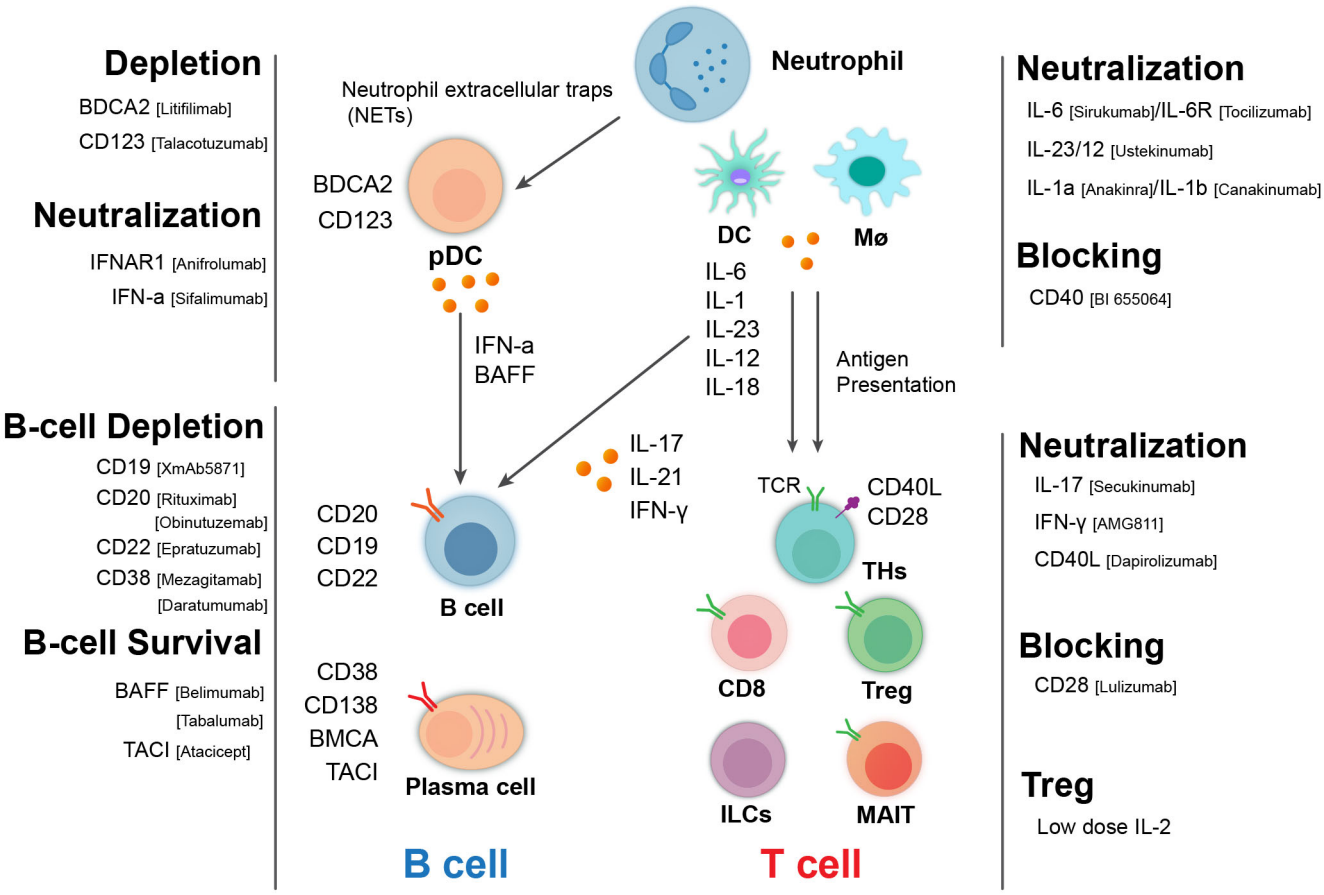
Immunological cellular and molecular players in lupus nephritis and targeted therapeutic biologics. Pathogenic cascade of LN involving many types of immune cells and related molecules. B cells produce autoantibody and cytokine and the activation of T cells. T cells including helper T cell subsets, cytotoxic T cells, and regulatory T cells (Tregs) can contribute to pathogenesis through different mechanisms. Myeloid cells including macrophages/monocytes, dendritic cells, and neutrophils can act upstream and downstream of the adaptive immune system to mediate disease processes. These cells and their interactions exacerbate kidney inflammation and damage in LN, leading to the clinical symptoms associated with the disease. Consequently, biologics targeting these cells and related molecules represent therapeutic options for LN.

A combination of hormonal, environmental, and genetic/epigenetic factors initiates the onset of SLE, as well as LN, through a chain reaction involving the activation of various immune cells, including myeloid cells/dendritic cells, neutrophils, T cells, and autoantibody-producing B cells (21) (**Figure 1**). In the following section, we summarize the key cellular and soluble players of the immune system in SLE and LN pathogenesis and related biologics.

## Autoreactive B cells, autoantibodies, and B-cell depletion therapies in LN

Autoantibody (AutoAb)-producing B cells play a central role in the pathogenesis of systemic lupus erythematosus (SLE) and lupus nephritis (LN). Notably, autoantibodies can be detected many years prior to the diagnosis of SLE (3). There has been intensive research aimed at understanding the mechanisms behind the development of autoantibody-producing B cells. Generally, the role of B cells in LN pathogenesis can be categorized as either autoAb-dependent or autoAb-independent.

B-cell activation often requires costimulation from T cells through membrane-bound and secreted molecules such as CD40L, OX40L, and various cytokines, including IL-4, IL-5, IL-6, and IL-13. A significant advancement in B-cell biology has been the identification of the B-cell-activating factor (BAFF or Blys) as a critical regulator of B-cell development, activation, survival, proliferation, and antibody production. BAFF interacts with three receptors expressed on B cells—the BAFF receptor, transmembrane activator and calcium modulator, and cyclophilin ligand interactor (TACI), and B-cell maturation antigen (BCMA)—to achieve its functional outcomes (22). In addition to BAFF, another member of the TNF family, a proliferation-inducing ligand (APRIL), plays a key role in plasma cell survival, isotype switching, and T-independent antibody responses (23, 24).

Autoantibodies, the main products of activated autoreactive B cells including plasma cells, can contribute to LN pathogenesis in several ways. A well-appreciated mechanism is that autoantibodies facilitate the formation of inflammatory immune complexes (ICs). These immune complexes can deposit in the kidneys, stimulating immune effector cells and affecting various kidney parenchymal cells, including epithelial cells, endothelial cells, and fibroblasts/myofibroblasts, leading to glomerular and tubular inflammation and fibrosis. In SLE, autoantibodies and immune complexes can recruit and activate neutrophils, leading to increased formation of neutrophil extracellular traps (NETs) and reduced NET degradation, thereby amplifying a neutrophil-centric inflammatory loop (24). Additionally, NETs can stimulate epithelial cells to secrete IL-36, and neutrophil elastase mediates the maturation and activation of IL-36α, -β, and -γ (25) as well as the activation of the IL-36 receptor antagonist (26).

B cells also contribute to LN pathogenesis through autoantibody-independent mechanisms. Notably, patients responding to B-cell depletion therapy (BCDT) often do not exhibit a corresponding decrease in autoantibody levels (27). Since B cells can modulate immune responses via antigen presentation, cytokine secretion (e.g., IL-17) (27, 28), and the initiation of tertiary lymphoid tissues (TLTs) (27), BCDT may confer clinical benefits by targeting these antibody-independent functions of B cells.

## B-cell targeting biologics in SLE and LN

Given the importance of B cells in the pathogenesis of systemic lupus erythematosus (SLE) and lupus nephritis (LN), in addition to B-cell depletion therapies (BCDTs), targeting the BAFF/APRIL/TACI pathways represents another B-cell-targeting therapeutic strategy for autoimmune diseases. Belimumab, a B-cell-targeted biologic that neutralizes soluble B lymphocyte stimulator (BLyS), is the first FDA-approved therapy for systemic lupus erythematosus (SLE). For refractory lupus nephritis (LN), rituximab—a chimeric anti-CD20 monoclonal antibody—is recommended as the standard second-line or salvage therapy according to guidelines from EULAR, KDIGO, and the Chinese Rheumatology Association (29). Given the complex pathogenesis of SLE, including LN, focusing solely on B cell targeting may not sufficiently address the symptoms experienced by patients with SLE (22).

## Dysfunction of dendritic cells and T cells in LN and relevant biologics under development

### Dysfunction of DCs in SLE

Dendritic cells (DCs) are professional antigen-presenting cells that are critical for the activation of T and B cells, initiating an immune response. DC dysfunction has long been considered a driver of the autoimmune response in SLE (30). Among the various types of DCs, plasmacytoid DCs (pDCs) have garnered significant attention due to their superior ability to produce type I interferon (IFN) (31). Additionally, pDCs produce BAFF and APRIL (32) and activate autoreactive B cells through cell–cell interactions (33). In the context of SLE pathogenesis, pDC activation can be triggered by neutrophil extracellular traps (NETs), which are present at higher levels in SLE patients (34). In lupus-prone mice, pDC dysfunction manifests in several ways: aged lupus-prone mice show pDC resistance to glucocorticoids (35), and pDCs from NZB mice have a longer lifespan than those from C57BL/6 mice, leading to elevated IFN-α levels in the former. Although pDCs are generally decreased in the peripheral blood of SLE patients, there is a concomitant infiltration of pDCs into nephritic kidneys (36).

Given the prominent role of pDCs in SLE pathogenesis, particularly as the main producers of type I IFN, inhibiting pDC activation and exploring pDC depletion have been investigated as potential treatments for human SLE by targeting surface receptors (BDCA and CD123) on human pDCs (36, 37). However, it remains unclear whether these interventions can benefit LN patients. Since pDCs rely on BCL-2 for survival, venetoclax, a BCL-2 inhibitor, has been trialed in women with SLE (38).

Targeting type I IFN has been extensively explored for SLE treatment, with biologics targeting either type I IFN itself or its receptor (IFNAR) (37). Anifrolumab is a monoclonal antibody that targets IFNAR, thereby blocking all type I interferons. In the TULIP trials, patients treated with anifrolumab demonstrated a higher proportion achieving an SLE Responder Index 4 (SRI-4) response at 52 weeks. Additionally, these patients experienced significant improvements in cutaneous (skin) and musculoskeletal (joint) manifestations. Some individuals also showed reductions in anti-dsDNA antibody levels and improvements in low complement levels (39). It is worth noting that anifrolumab has not been reported to be used for the treatment of lupus nephritis (LN).

### Dysfunction of T cells and target biologics

The activation of B cells, including autoreactive B cells, often requires help from T cells. The following section will briefly discuss the role of T cells in the pathogenesis of systemic lupus erythematosus (SLE).

#### CD4^+^T cells

CD4^+^T cells and their functionally biased subsets significantly influence various autoimmune responses through collaboration and division of labor. Different subsets of CD4^+^T cells contribute to the pathogenesis of SLE in distinct ways (40). Interferon-gamma (IFN-γ) produced by Th1 cells promotes B cell class switching and stimulates the production of pathogenic autoantibodies by inducing the aberrant activation of T follicular helper (Tfh) cells (41). Th2 cells secrete cytokines such as IL-4, IL-5, and IL-13, which are well documented for their roles in regulating autoantibody production and other mechanisms relevant to SLE pathogenesis. Abnormalities in T helper (Th) cells in SLE also extend to Th22 and Th9 subsets (40).

Aberrant activation of Tfh cells plays a crucial role in the production of autoantibodies. Dysregulation of Tfh cells in autoimmune diseases includes altered transcription factor expression, cytokine and chemokine production, co-stimulation, metabolic changes, exhaustion, and trafficking (42). Consequently, molecules related to Tfh cells have been explored as therapeutic targets for SLE. In lupus-prone mice, blocking ICOS/ICOS-L interactions, OX40L, CD40L, or neutralizing IL-21 has been shown to reduce disease progression. Biologics targeting these molecules are currently under evaluation in clinical studies, including AMG 557 for ICOS-L blockade (NCT02391259) and BI 655064 for CD40L inhibition (NCT02770170) (43, 44).

#### T regulatory cells

T regulatory cells (Tregs) are primarily classified as CD4^+^T cells characterized by a high expression of membrane CD25 and intracellular forkhead box P3 (Foxp3). However, accumulating evidence suggests the existence of non-CD4^+^Tregs as well. Tregs can directly suppress the production of autoantibodies (autoAbs) by B cells in systemic lupus erythematosus (SLE) (45). The adoptive transfer of Treg cells has been shown to delay disease progression in mouse models of lupus (46).

As summarized in a comprehensive review, dysregulation of Tregs in SLE may not simply be reflected as a reduction in Treg numbers in circulation. A disturbed balance between effector T cells and Tregs occurs instead due to a significant increase in autoreactive T cells or reduced Treg function (47). A recent study demonstrated that autoantigen-specific Sm-Tregs potently suppress inflammatory responses *in vitro*and inhibit disease progression in a humanized mouse model of lupus nephritis (48). Clinical trials involving Treg therapy have been conducted for various autoimmune disorders, organ transplantation, and other inflammatory diseases (49). In addition to cell therapy, biologics targeting Tregs have also been explored for treating autoimmune diseases, including lupus nephritis. Non-Fc receptor-binding anti-CD3 monoclonal antibodies have been shown to increase Treg populations and have been delivered in various formats to induce immune tolerance in clinical trials (50, 51). Furthermore, specific unique anti-CD4 antibodies have been found to activate Tregs (52). Tregalizumab has entered clinical studies for psoriasis and rheumatoid arthritis, demonstrating promising clinical effects (53). Additionally, clinical studies have shown that low-dose IL-2 can expand Tregs and improve outcomes in lupus nephritis (54, 55). Overall, Treg-targeting interventions are still in the early stages as effective treatments for lupus nephritis.

#### CD8^+^T cells

In addition to CD4^+^helper T cells, CD8+ T cells are also believed to play a role in the pathogenesis of SLE and LN (21, 56). CD8^+^T cells from the peripheral blood of SLE patients exhibit reduced effector function, which is attributed to decreased granzyme B and perforin production (57). Furthermore, IL-17-producing double-negative T cells (lacking a surface expression of both CD4 and CD8) are derived from autoreactive CD8^+^T cells in tissues expressing autoantigens (58, 59). A recent study revealed that the effector function of CD8^+^CD27^+^CXCR3^−^T cells is overactive in active SLE compared with healthy controls and patients in remission, and this overactivity is positively associated with clinical SLE activity (60).

## IL-17 and TH17 in the pathogenesis of lupus nephritis

While numerous cellular and molecular players contribute to the pathogenesis of lupus nephritis (LN), this section will focus on the role of the IL-17 family. Various members of the IL-17 family have been shown to impact chronic kidney diseases, including lupus nephritis (10). Despite several case reports indicating promising therapeutic benefits with IL-17 antagonists, a recent phase III trial evaluating the efficacy of secukinumab in patients with active lupus nephritis was terminated following an interim analysis for futility, with no safety concerns identified (5). Despite the disappointing results, the potential of IL-17 antagonists in the treatment of lupus nephritis (LN) cannot be entirely dismissed, given the strong association between IL-17 and SLE/LN as well as encouraging findings from multiple case reports. It is hypothesized that combination approaches may enhance the efficacy of IL-17 antagonists in managing LN. Such combination therapies could involve pairing IL-17 antagonists with other immunomodulators, including corticosteroids, antimalarials (e.g., hydroxychloroquine), or immunosuppressants (e.g., mycophenolate mofetil and cyclophosphamide) used in SLE treatment. Moreover, they may be combined with JAK/STAT inhibitors, B-cell modulators (e.g., rituximab, belimumab), or through co-blockade of other pro-inflammatory cytokines, including those discussed above. In this review, the authors primarily explore the potential therapeutic benefits and underlying mechanisms of co-blockade involving the IL-17 and IL-36 cytokine families, focusing on their roles in inflammatory processes and their potential to synergistically modulate immune responses in various inflammatory and autoimmune conditions.

### IL-17 family

The IL-17 family consists of six distinct members (IL-17A to IL-17F) that exert their physiological effects through interactions with IL-17 receptors (IL-17RA to IL-17RE). Among these, IL-17A (hereafter referred to as IL-17) has been extensively studied and, along with IL-17F, mediates its biological functions by binding to IL-17RA and IL-17RC. IL-17 is primarily produced by CD4^+^T cells known as T helper 17 (Th17) cells (61). An increase in Th17 cells in lupus nephritis (LN) has been observed in the peripheral blood mononuclear cells of LN patients (61, 62). However, kidney CD4^+^T cell clusters from LN patients have also been reported, with unclear associations to Th1 or Th17 signatures (21). Nonetheless, IL-17 can originate from various cellular sources, including CD8^+^T cells, γδ T cells, innate lymphoid cells (ILCs), natural killer (NK) cells, invariant NK T cells, mucosal-associated invariant T cells, mast cells, and Paneth cells. Furthermore, while T cell receptor (TCR) activation is crucial for IL-17 production by conventional T cells, innate immune cells rely on inflammatory cytokines—particularly IL-1β and IL-23—to drive IL-17 secretion (63).

### The role of IL-17 in the pathogenesis of lupus nephritis

In lupus nephritis, IL-17A is implicated in multiple stages of disease progression, including modifying the structure and function of specialized renal cells, fostering an inflammatory environment, and contributing to recurrent tissue damage and ineffective repair processes, ultimately leading to renal fibrosis and functional decline. The following section outlines existing research on the impact of IL-17A on distinct renal cell types and compartments (63).

#### Impact on podocytes

IL-17 promotes podocyte motility. Experiments have demonstrated that Th17 cells may release factors that enhance podocyte movement, resulting in cytoskeletal changes and increased permeability, potentially leading to cellular dysfunction and apoptosis (64).

#### Influence on mesangial cells

IL-17 stimulates mesangial cells to release chemokines. When mesangial cells are stimulated with IL-17A or IL-17F, they produce and release chemokines CCL2 and CXCL2 in a MAPK-dependent manner, exhibiting dose- and time-dependent characteristics (65). In accelerated models of diabetic nephropathy, the presence of IL-17 correlates with glomerular basement membrane thickening, and the inhibition of IL-17A with antibodies mitigates this effect (66). Furthermore, in models of anti-glomerular basement membrane glomerulonephritis, the Th17/IL-17 pathway drives inflammation and autoantibody-induced kidney injury, with inhibition or knockout of IL-17-reducing pro-inflammatory cytokines (67).

#### Effects on renal tubular epithelial cells

IL-17 enhances inflammatory and fibrotic mechanisms in renal tubular epithelial cells. Numerous studies have shown that exposure to IL-17 leads to the upregulation of various mediators, including cytokines, chemokines, and growth factors such as IL-6, IL-1β, and TNF-α. In lupus nephritis models, IL-17 and IFN-α stimulate tubular epithelial cells, resulting in a significant increase in CCL2 expression, which is chemotactic for dendritic cells and macrophages. In IL-17RA-deficient mice, renal infiltration of macrophages was markedly reduced despite no significant changes in systemic responses (7). Additionally, in autoimmune glomerulonephritis models, IL-17 stimulation of tubular epithelial cells increased the mRNA expression of chemokines such as CXCL1, CXCL2, and CXCL8, which attract monocytes and neutrophils (68). Consequently, IL-17 activation of tubular epithelial cells can recruit dendritic cells and macrophages, significant sources of TGF-β, thus promoting renal fibrosis (69). This highlights IL-17’s crucial role in driving tubular epithelial-mediated immunopathogenesis in lupus nephritis.

#### Influence on neutrophil dynamics, renal fibrosis, and other pathologies

Stimulation of renal tubular epithelial cells by IL-17A influences neutrophil dynamics, leading to the production of granulocyte colony-stimulating factor (G-CSF) in a dosage- and time-dependent manner. IL-17A and IL-17F can induce the expression of chemokines CXCL1 and CXCL5 in kidney tubular cells and mesangial cells, thereby facilitating significant neutrophil recruitment and subsequent renal tissue damage (68, 70, 71). Overall, IL-17 serves as a potent mediator of neutrophil-induced injury, promoting renal differentiation and neutrophil recruitment (71). In obstructive uropathy models, IL-17A promotes renal fibrosis by increasing TGF-β1 expression. It stimulates fibronectin production in renal cells via the TGF-β/Smad pathway, which can be inhibited by anti-TGF-β1 antibodies or TGF-β1 receptor inhibitors (72). Furthermore, studies indicate that IL-17A activates myofibroblasts and promotes extracellular matrix deposition, with mice lacking IL-17 being protected from subsequent obstructive fibrosis (73).

In experimental studies on hypertension and angiotensin II-induced fibrosis, the application of specific antibodies to block IL-17A or IL-17RA resulted in a significant reduction in the fibrotic marker TGF-β1 (74, 75). Conversely, the renal anti-fibrotic properties of various drugs have been linked to the suppression of IL-17 levels (76–78). Multiple studies have demonstrated that IL-17A can induce epithelial–mesenchymal transition (EMT) in renal tubular epithelial cells. One such study indicated that IL-17A stimulated cell proliferation and extracellular matrix secretion in cultured cells, leading to a shift from an epithelial to a mesenchymal phenotype via a TGF-β1-dependent pathway (79).

IL-17A is also implicated in thrombotic events and vascular dysfunction. Thrombotic microangiopathy is recognized as a contributing factor to poor prognosis in lupus kidney biopsies, characterized by endothelial damage and thrombosis. Although preliminary investigations are lacking, it is crucial to evaluate the potential role of IL-17A in thrombotic occurrences among lupus patients. Experimental research in psoriatic models has demonstrated that IL-17A facilitates thrombotic events and vascular dysfunction. Additionally, a study involving endothelial cells from individuals with rheumatoid arthritis revealed that IL-17, in combination with TNF-α, induced coagulants and a pre-thrombotic phenotype, surpassing the inflammatory state (79, 80).

IL-17 also affects blood pressure regulation. Hypertension is a recognized manifestation of renal involvement in lupus, and its association is a significant prognostic factor in lupus nephritis. A study found that IL-17A drives angiotensin II-induced hypertension by increasing renal sodium reabsorption (via the upregulation of ENaC and NCC transporters) and promoting kidney injury through inflammation, oxidative stress, and fibrosis. Blocking IL-17A reduced the blood pressure and renal damage, suggesting its therapeutic potential in hypertensive kidney disease (81). In a separate experimental investigation, IL-17A emerged as a crucial factor in arteriole vessel remodeling. Elevated levels of IL-17A contribute to increased blood pressure by promoting arterial remodeling and stiffness. Furthermore, the administration of antihypertensive drugs effectively reduces blood pressure without affecting the underlying structural alterations. Conversely, in SLE mice models, inhibition of IL-17A using antibodies results in decreased blood pressure and mitigated vascular remodeling, indicating a lasting impact on vascular architecture beyond mere hemodynamic changes (82).

Finally, IL-17A has a systemic effect on the generation of renal autoAbs. Beyond its localized effects on various kidney cells, IL-17A plays a role in the production of B-cell autoantibodies, as evidenced by studies in autoimmune models. These studies have shown that IL-17 drives the formation of autoreactive germ centers (GC) and that B-cell development and humoral responses are diminished in mice lacking the IL-17 receptor (83). Moreover, experimental research has demonstrated that IL-17 enhances the production of anti-double-stranded DNA antibodies and promotes the survival of plasma cells (84).

#### Clinical evidence of IL-17A associated with LN

Albuminuria, hematuria, and anemia are clinical manifestations significantly correlated with serum IL-17 levels. Specifically, the baseline concentrations of IL-17 have been positively linked to the severity of albuminuria. In a study involving 15 patients undergoing kidney biopsy, the presence of IL-17^+^TCR^+^cells in renal infiltrates was positively associated with hematuria in lupus nephritis as determined through laser microdissection techniques. Additionally, elevated serum levels of IL-17 and IL-6 were found to correlate with anemia in another study (85).

A study examining the relationship between severity scores and histological activity in systemic lupus erythematosus revealed significant associations between Th17 cell frequency, serum IL-17 levels, TWEAK (TNF-related weak inducer of apoptosis) levels, and the nephritis activity index. Furthermore, IL-17 concentration was positively correlated with erythrocyte sedimentation rate (ESR), systemic lupus erythematosus disease activity index (DAI) score, and antinuclear antibody (ANA) titer at baseline (86). Another study demonstrated significantly higher IL-17 levels in patients with severe lupus nephritis compared with controls, with urinary IL-17 levels increasing alongside disease severity (87).

In a study involving 52 patients with active lupus nephritis who underwent kidney biopsy at baseline and after receiving immunosuppressive therapy, individuals who did not respond to steroid treatment exhibited higher levels of IL-17 expression in inflammatory cells infiltrating kidney tissue. Conversely, after 6 months of treatment, there was a significant decrease in IL-17 levels among patients with active lupus nephritis (88).

### IL-17 antagonism in lupus nephritis in clinical settings

Studies investigating the pathological roles of IL-17 in systemic lupus erythematosus (SLE) suggest that IL-17A is a promising therapeutic target for this condition. The cytokines and receptors within the IL-17 family possess distinctive molecular structures that differentiate them from other protein families, making them attractive candidates for therapeutic intervention (89, 90). Current Th17-targeted therapies primarily involve monoclonal antibodies directed against IL-17, IL-23, and their receptors. Notable IL-17A antagonists include secukinumab and ixekizumab, while bimekizumab targets both IL-17A and IL-17F. Brodalumab inhibits IL-17 by binding to the IL-17 receptor (IL-17R) (91). Additionally, upstream neutralization of IL-23 can be achieved with guselkumab, risankizumab, tildrakizumab, and ustekinumab. These modalities have been widely used for the treatment of psoriasis (PsO), psoriatic arthritis (PsA), and spondyloarthritides (SpA) and even inflammatory bowel diseases (IBD) (92, 93). Given the success of anti-IL-17 therapies in treating these autoimmune diseases, the potential for similar approaches in SLE to reduce disease activity has also been investigated (94).

Ongoing clinical trials, SELUNE and ORCHID-LN, are evaluating secukinumab and guselkumab in lupus nephritis. The SELUNE study (NCT04181762) is a phase III randomized, double-blind trial designed to assess the efficacy and safety of secukinumab in conjunction with standard care therapy for individuals with active lupus nephritis. However, the study was prematurely halted by the sponsor following a futility analysis (9). The ORCHID-LN trial investigates the safety and efficacy of guselkumab in individuals with active lupus nephritis, comparing its addition to standard care with a placebo combined with standard care. This study was also prematurely terminated by the sponsor due to difficulties in participant enrollment.

Although numerous IL-17-related biologics have been evaluated in systemic lupus erythematosus and lupus nephritis, most phase II trials have not yielded statistically significant results that meet the regulatory criteria. Nonetheless, there are documented case reports indicating the successful use of IL-17 antagonism in lupus nephritis (**Table 1**).

**Table 1:** Case reports on IL-17 antagonism in SLE/LN.

Patient	Age	Gender	Conditions	Treatment	Outcome	Ref.
1	41	Male	Severe psoriasis, hypertrophic discoid lupus	Ustekinumab 45 mg SC (d1, w4, w16); 90 mg SC at w20 (4th dose)	Complete clearance of psoriasis plaques; moderate improvement in lupus plaques (clinical assessment)	(95)
2	52	Female	28-year history of disfiguring DLE	Ustekinumab 45 mg + methotrexate + intralesional corticosteroids (switched to monotherapy)	Sustained improvement after 48 months; no adverse events; stable skin lesion scores (physician global assessment)	(96)
3	62	Female	Psoriasis vulgaris, refractory lupus nephritis	Secukinumab (dose not specified in original report)	Notable improvement in clinical parameters (reduced proteinuria, stable eGFR)	(97)
4	N/A	Male	PsA, SLE	Secukinumab (dose not specified in original report)	Marked reduction in serum IL-6; undetectable IL-17; no SLE exacerbation noted	(98)
5	N/A	Female	Refractory lupus nephritis	Secukinumab (dose not specified in original report)	Significant improvement in clinical/biological parameters; complete renal response (proteinuria <0.5 g/24h, stable eGFR)	(99)
6	N/A	N/A	Exacerbation of DLE after psoriasis treatment	Secukinumab 150 mg SC q4w for 2 years	Significant improvement in psoriasis/PsA; progressive DLE lesions (increased lesion area, inflammation score)	(100)
7	23	Female	Psoriasis, newly diagnosed SLE	Secukinumab 300 mg SC	SLEDAI score reduction from 12 to 6; stable renal function (follow-up ongoing)	(101)
8	68	Male	Psoriatic arthritis, suspected DILE	Secukinumab (discontinued); treated with enoxaparin, prednisolone, etc.	Clinical/laboratory remission within 1 month; persistent proteinuria (653 mg/24 h) resolved with mycophenolate mofetil	(102)

### Co-blockade of IL-17 and IL-36 pathways in LN

**Figure 2:**
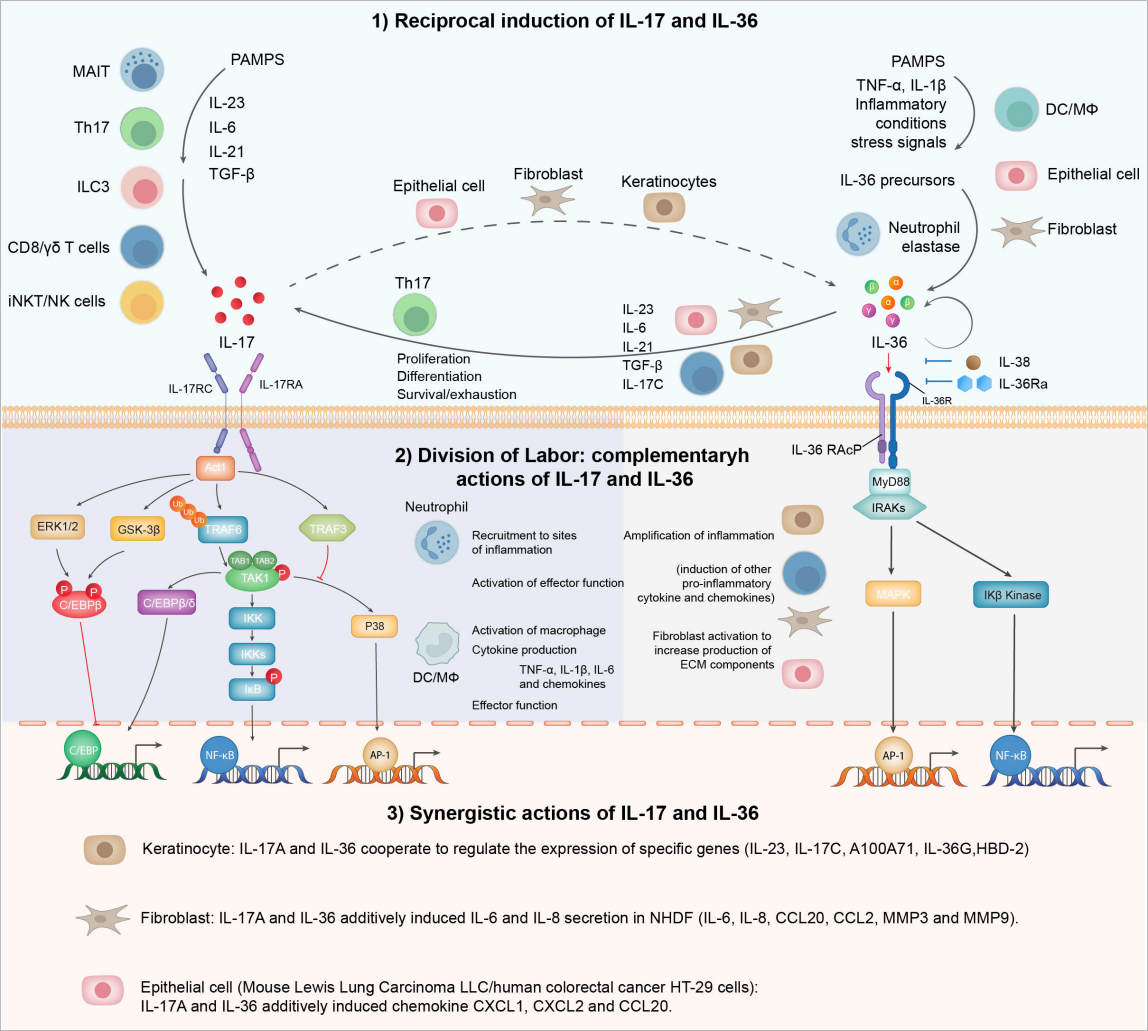
IL-17 and IL-36 inflammatory axis: from division of labor to synergy. IL-17 and IL-36 engage in a reciprocal relationship where the presence of one cytokine enhances the production of the other. This interaction amplifies inflammatory responses and may play a significant role in autoimmune conditions. Functionally, IL-17 and IL-36 are pivotal in immune regulation and inflammation, each with distinct roles: IL-17 primarily recruits neutrophils and promotes the release of other pro-inflammatory cytokines, while IL-36 is involved in skin inflammation and the activation of epithelial cells and fibroblasts. These cytokines exhibit a synergistic interplay, particularly evident in epithelial cells and fibroblasts.

Most clinical trials with IL-17 antagonists have not demonstrated statistically significant outcomes for lupus nephritis (LN) patients despite encouraging signals from several case reports. Combination therapy targeting additional molecules or pathways might be beneficial for complex diseases like lupus nephritis. Given the documented association of the IL-36 pathway with systemic lupus erythematosus (SLE), reciprocal regulation, and the synergistic action between IL-17 and IL-36 in inflammation and fibrosis (**Figure 2**), we discuss the rationale for co-blockading IL-17 and IL-36 pathways in lupus nephritis.

### IL-36 family

The IL-36 cytokines, which include three agonists—IL-36α, IL-36β, and IL-36γ—and two antagonists—IL-36Ra and IL-38—belong to the IL-1 family. IL-36 exhibits pro-inflammatory characteristics and plays a crucial role in immune cell activation and antigen presentation. The signaling pathways initiated by IL-36 agonists interact with IL-1RAcP and IL-36R, leading to the recruitment of MyD88, IRAK4, and TRAF6. This cascade ultimately activates the NF-κB and MAPK pathways, facilitating pro-inflammatory signaling. In contrast, IL-36 antagonist signals bind to IL-1RAcP and IL-36R, inhibiting NF-κB and MAPK signaling, thereby promoting anti-inflammatory responses (103).

The skin serves as the primary site for IL-36 cytokine expression, with several studies indicating their significant involvement in the pathogenesis of various skin diseases. Monoclonal antibodies targeting IL-36R have been approved for treating generalized pustular psoriasis, and research is ongoing to investigate the role of IL-36 signaling in other autoimmune conditions (104).

### Elevated levels of IL-36 in SLE and disease association

The association of IL-36 with systemic lupus erythematosus (SLE) has been recognized for some time. In a study involving 43 SLE patients and 16 normal control (NC) subjects, the plasma concentrations of IL-36α and IL-36γ were significantly elevated in active SLE patients compared with NC. Notably, the plasma levels of IL-36α and IL-36γ correlated positively with SLE disease activity. Additionally, the proportions of circulating IL-36R-positive CD19^+^B lymphocytes among total B lymphocytes and PBMCs were significantly higher in active SLE patients. Upon *ex vivo*stimulation with IL-36α and IL-36γ, the production of IL-6 and CXCL8 was significantly increased in SLE patients compared with NC, suggesting that IL-36α may act as a pathogenic factor in SLE (105).

Another study involving 72 SLE patients and 63 healthy controls in China also found significantly increased serum levels of IL-36α and IL-36γ along with decreased serum IL-36Ra levels in SLE patients compared with healthy controls. Active SLE patients (SLEDAI (Systemic Lupus Erythematosus Disease Activity Index) score ≥5) exhibited significantly higher serum levels of IL-36α and IL-36γ than the inactive patients (SLEDAI score ≤4). Moreover, these levels were strongly correlated with SLEDAI scores and complement C3 levels. Notably, SLE patients with arthritis had significantly elevated serum IL-36α and IL-36γ levels compared with those without arthritis (106). A recent study extended the investigation to IL-36β and IL-36R, finding that these proteins were expressed in immune cells as well as epithelial cells of SLE patients, linking them to specific disease features (107). In another study, significantly increased serum levels of IL-36α and pentraxin 3 were detected in both active (*P*= 0.000 for both) and inactive SLE patients (*P*= 0.003 and *P*= 0.001, respectively) compared with normal controls. Nevertheless, active SLE patients had significantly higher IL-36α levels compared with inactive patients (108). Similar conclusions were reached when evaluating the IL-36α mRNA levels. In a separate study involving 49 SLE patients and 40 healthy controls, IL-36α mRNA was significantly higher in SLE patients, with fold changes indicating increased expressions in those with moderate to high disease activity (SLEDAI >5) compared with those with mild activity (SLEDAI ≤5) (109).

In the context of lupus nephritis (LN), the urinary levels of IL-36 cytokines were examined in a study involving 196 SLE patients—comprising 97 with active LN, 42 with inactive LN, and 57 with active lupus without renal involvement—as well as 25 healthy subjects (110). Although the cytokine levels in urine were generally low, the urinary IL-36γ levels were significantly elevated in SLE patients compared with healthy controls. Patients with active LN exhibited markedly higher IL-36γ levels than those without renal involvement, and these levels showed a moderate correlation with renal SLEDAI scores. Notably, the urinary IL-36γ levels decreased significantly after 3 months of immunosuppressive therapy in patients with active LN (110).

IL-38, an antagonist of the IL-36 family, has been implicated in various autoimmune and inflammatory diseases, predominantly functioning as an anti-inflammatory cytokine (111). In the context of lupus nephritis, IL-38 levels were found to be significantly higher in samples from systemic lupus erythematosus (SLE) patients—particularly those with active disease—compared with healthy controls. Furthermore, the presence of IL-38 was linked to an increased risk of renal lupus.

Peripheral blood mononuclear cells (PBMCs) treated with IL-38 siRNA (small interfering RNA) produced up to 28-fold more of the pro-inflammatory mediators IL-6, CCL2, and APRIL than control siRNA-transfected cells when stimulated with Toll-like receptor agonists, suggesting a protective role for IL-38 (112, 113). Additionally, both the mRNA and protein levels of IL-38 in the peripheral blood of SLE patients were observed to decrease (114).

It is worth noting that a recent study also evaluated both IL-36 and IL-17 in SLE patients. Consistent with previous findings, the IL-36α levels were significantly higher in SLE patients, particularly in those with an active disease. Furthermore, the serum IL-17 levels were elevated in SLE patients, with a positive correlation observed between IL-36α and IL-17 levels. Importantly, patients with lupus nephritis had higher serum IL-36α levels compared with those without LN. This study also noted that patients receiving glucocorticoid treatment had lower IL-36α levels than those not receiving such treatment (115).

### IL-36 in disease models of chronic kidney diseases

While the role of IL-36 has not been directly examined in the context of LN, an increased expression of IL-36α has been reported in renal tubular epithelial cells from a mouse model of unilateral ureteral obstruction (UUO). Compared with UUO-treated wild-type mice, IL-36 knockout (IL-36-/-) mice exhibited a markedly reduced NLRP3 inflammasome activation as well as decreased macrophage and T cell infiltration in the kidneys and T cell activation in the renal draining lymph nodes, leading to diminished formation of renal tubulointerstitial lesions (TILs). Notably, *in vitro*studies demonstrated that recombinant IL-36α facilitated NLRP3 inflammasome activation in renal tubular epithelial cells, macrophages, and dendritic cells and enhanced dendritic cell-induced T cell proliferation and Th17 differentiation. Furthermore, the deficiency of IL-23, which was diminished in IL-36R knockout UUO mice, also reduced renal TIL formation in UUO models (116).

In a related study using the UUO model, IL-36α was found to be overexpressed in injured distal tubules (DTs). Importantly, IL-36α expression significantly correlated with the progression of tubulointerstitial cell infiltration and tubular epithelial cell death in UUO kidneys. The IL-1RL2 receptor for IL-36α localized to podocytes, proximal tubules, and DTs in healthy kidneys, but in UUO kidneys IL-1RL2 was expressed in interstitial cells, platelets, and extended primary cilia of DT epithelial cells. Stimulation with IL-36α promoted the production of IL-6 and Prss35, an inflammatory cytokine and collagen remodeling-associated enzyme, respectively, in cultured NIH3T3 (the embryonic mouse fibroblast cell line) fibroblasts. IL-36α knockout (KO) mice exhibited milder features of kidney injury compared with wild-type (WT) mice in UUO models (117).

A few studies have conducted a detailed dissection of the IL-36 family’s role in lupus nephritis. In MRL/MpJ-Faslpr/lpr mice, IL-36R deficiency resulted in reduced glomerular lesions, particularly mesangial matrix expansion, with significant amelioration observed in both sexes of IL-36R^-/-^mice compared with WT mice. IL-36R deficiency had minimal effects on the indices of immune abnormalities, renal function, and serum anti-dsDNA antibody levels (118).

Notably, recombinant IL-38 was found to attenuate clinical severity in the MRL/lpr mouse model (119). In a pristane-induced lupus mouse model, the deficiency of IL-38 exacerbated inflammation, upregulated inflammatory cytokines and autoantibodies, and led to severe pathological changes in the kidneys. The administration of recombinant murine IL-38 to pristane-treated IL-38-/- mice improved their renal histopathology (120). Treatment with human recombinant IL-38 protein *in vitro*reduced the levels of IKKα/β, NF-κB, and TNF-α and decreased the anti-dsDNA antibodies in PBMCs from SLE patients. Additionally, kidney function—reflected by creatinine and blood urea nitrogen levels—along with anti-dsDNA antibodies, complement C3, and urinary protein levels decreased following treatment with IL-38 protein in MRL/lpr lupus mice. However, IL-38 protein treatment also induced mild hyperplasia of glomerular mesangial cells and lymphocyte infiltration (114).

### Cross-talk between IL-36 to IL-17 in the context of inflammation

#### Impact of IL-36 on IL-17 and Th17

IL-36 stimulates the production of IL-17C and IL-23 by adult normal human epidermal keratinocytes while also self-amplifying its own production (116). Inhibition of the IL-36 receptor by IL-36Ra has been shown to reduce *Aspergillus*-induced IL-17 and IFN-γ levels (121). In patients with generalized pustular psoriasis (GPP) harboring mutations in the IL-36 antagonist IL36RN, CD4^+^T cells in both blood and skin lesions exhibited intense hyperproliferation and production of IL-17 (122).

IL-36 is also known to promote the differentiation and function of human Th17 cells. Anti-IL-36α treatment has been shown to alleviate the Th17 response in a mouse model of allergic rhinitis, resulting in reduced symptoms, decreased Th17 cell infiltration, and downregulated expression of Th17 cytokines (123). In an imiquimod-induced psoriasis-like dermatitis model, keratinocyte-specific IL-36R deficiency led to the reduced induction of IL-23, IL-17, and IL-22 at lesion sites. Additionally, IL-36γ is known to induce the expression of IL-17C by keratinocytes (124).

IL-17C, a member of the IL-17 family, is primarily produced by epithelial cells. Neutralization of IL-17C has been shown to mitigate albuminuria, mesangial matrix accumulation, and podocyte loss. Furthermore, IL-17C neutralization significantly repressed the expression of downstream pro-inflammatory cytokines, inflammatory cell infiltration, and Th17/IL-17A activation in both acute and chronic kidney injury models (125). Correspondingly, IL-38 was found to suppress the expression of IL-23R and IL-17A in circulating PBMCs as well as IL-6 and IL-8 in resident-activated orbital fibroblasts (126).

#### Impact of IL-17 on IL-36

The IL-36 cytokines can be induced in cultured primary human keratinocytes (KCs) by IL-17A (127). IL-17A also activates the expression of IL-36 cytokines in keratinocyte monolayer cultures (128). Specifically, IL-17A *in vitro*induced the expression of IL-36β and IL-36γ at both gene and protein levels in HaCaT (high sensitivity of human epidermal keratinocytes) cells (129). This indicates that IL-17A promotes the production of IL-36 by human keratinocytes. Furthermore, IL-36, in conjunction with IL-17A, effectively activated human dermal microvascular endothelial cells (HDMECs), which express both IL-17 and IL-36 receptors (130). The direct interaction of IL-17 and IL-36 in kidney injury remains to be elucidated.

### Synergistic action of IL-36 and IL-17 in regulating target genes

Molecular analyses have demonstrated the strong cooperative effects of IL-17A and IL-36 cytokines in regulating target genes, including CCL-20, IL-8, and antimicrobial peptides (AMPs) (128). Adult normal human epidermal keratinocytes were stimulated for 24 h with recombinant IL-36 and IL-17. Both IL-36α and IL-36γ self-amplified their mRNAs and synergistically enhanced the mRNA and protein levels when combined with IL-17A. Notably, IL-17C was significantly increased by IL-36 alone or in synergy with IL-17A. Additionally, combinations of IL-17A and IL-36γ significantly elevated the expressions of IL12B and IL23A. We recently reported that IL-17 and IL-36 work together to amplify the expression of pro-inflammatory and pro-fibrotic genes in normal human dermal fibroblasts (NHDF). Co-blocking IL-17 and IL-36 is more effective at inhibiting the production of IL-6 and IL-8 in NHDF stimulated by IL-17A and IL-36 compared with using two separate monoclonal antibodies (131).

It is clear that the IL-17 and IL-36 signaling pathways have both overlapping and distinct roles in various inflammatory contexts. However, the interaction between these two cytokines in the pathogenesis of chronic kidney diseases, including lupus nephritis (LN), remains to be fully explored. Given that a combination of approaches may be necessary to harness the potential of IL-17 antagonism in LN, it is important to determine whether co-targeting both pathways could enhance disease management, leading to immunological improvements and overall better disease outcomes.

## Conclusion/perspective

Several biologics targeting B cells and type I interferon have been approved for the management of systemic lupus erythematosus (SLE), including lupus nephritis (LN). Additionally, strategies targeting regulatory T cells (Tregs) and plasmacytoid dendritic cells (pDCs) have been investigated in clinical studies. Due to extensive research, IL-17 and cytokines that regulate IL-17-producing cells have also been targeted in the treatment of LN. However, randomized controlled trials involving anti-IL-17 antibodies have shown limited efficacy. One potential reason for this limited effectiveness may be the need to target multiple pathways across different stages of the disease, involving various immune and kidney resident cells, given the complexity of LN. This study proposes that the proinflammatory cytokine IL-36 may serve as a valuable therapeutic partner to IL-17 antagonists in the treatment of LN. Given the overlapping and synergistic roles of IL-36 and IL-17 in promoting inflammation and tissue damage within the renal microenvironment, combined targeting of these cytokines could potentially enhance anti-inflammatory effects, improve clinical outcomes, and offer a novel, synergistic approach to managing this complex autoimmune condition.
